# A Histomorphological Pattern Analysis of Pulmonary Tuberculosis in Lung Autopsy and Surgically Resected Specimens

**DOI:** 10.1155/2016/8132741

**Published:** 2016-03-21

**Authors:** Mamta Gupta, Flora D. Lobo, Deepa Sowkur Anandarama Adiga, Abhishek Gupta

**Affiliations:** ^1^Department of Pathology, Subharti Medical College, Swami Vivekananda Subharti University, Meerut, Uttar Pradesh 250002, India; ^2^Department of Pathology, Kasturba Medical College, Mangalore, Manipal University, Karnataka 575001, India; ^3^Department of Medicine, Subharti Medical College, Swami Vivekananda Subharti University, Meerut, Uttar Pradesh 250002, India

## Abstract

*Background*. Tuberculosis (TB) is a major cause of morbidity and mortality globally. Many cases are diagnosed on autopsy and a subset of patients may require surgical intervention either due to the complication or sequelae of TB.* Materials and Methods*. 40 cases of resected lung specimens following surgery or autopsy in which a diagnosis of pulmonary tuberculosis was made were included. Histopathological pattern analysis of pulmonary tuberculosis along with associated nonneoplastic changes and identification of* Mycobacterium tuberculosis* bacilli was done.* Results*. The mean age of diagnosis was 41 years with male predominance (92.5%). Tuberculosis was suspected in only 12.1% of cases before death. Seven cases were operated upon due to associated complications or suspicion of malignancy. Tubercular consolidation was the most frequent pattern followed by miliary tuberculosis. The presence of necrotizing granulomas was seen in 33 cases (82.5%). Acid fast bacilli were seen in 57.5% cases on Ziehl-Neelsen stain.* Conclusion*. Histopathology remains one of the most important methods for diagnosing tuberculosis, especially in TB prevalent areas. It should be considered in the differential diagnosis of all respiratory diseases because of its varied clinical presentations and manifestations.

## 1. Introduction

Tuberculosis (TB) continues to remain one of the most pressing health problems in India. India is the highest TB burden country in the world, accounting for one-fifth of the global incidence with an estimated 1.96 million cases annually [1].

Tuberculosis is endemic in many parts of the world and causes many deaths. The most common form is pulmonary tuberculosis, characterized pathologically by necrotizing granulomas, associated pneumonia, and a great propensity for fibrosis and dystrophic calcification [2].

Due to the stigma associated with this disease, in many cases of active TB, either there is a delay in diagnosis and treatment leading to unrestricted exposure of bacilli to environment or the patient fails to take adequate treatment causing multidrug resistance. A subset of patients may require surgical intervention due to the associated complications. The prevalence of tuberculosis is high in the Indian subcontinent, and cases may not be diagnosed until after an autopsy is performed [3–6].

Various complications of pulmonary tuberculosis significantly contributing to morbidity and mortality of patients include hemoptysis, posttubercular bronchiectasis, aspergilloma, tubercular endobronchitis and tracheitis, spontaneous pneumothorax, scar carcinoma, disseminated calcification in lung, pulmonary function changes, obstructive airway disease, and secondary pyogenic infections. Systemic manifestations in the form of secondary amyloidosis and chronic corpulmonale may also occur [7].

The hallmark of* Mycobacterium tuberculosis* infected tissue is necrotizing granulomatous inflammation, composed of epithelioid histiocytes surrounding a central necrotic zone, and can be accompanied by a variable number of multinucleated giant cells and lymphocytes. Nonnecrotizing granulomas can be present as well. The major function of epithelioid histiocytes is to contain the infection to a localized area, thus avoiding bacterial spread to surrounding healthy tissues and to other organs, and to concentrate the immune response to a limited infectious area [8].

TB is a major cause of morbidity and mortality globally. Many cases may go undetected and are diagnosed only after an autopsy is performed. Another subset of cases present with various complications or sequelae of the disease process. The present study was undertaken to evaluate the histopathological spectrum of pulmonary tuberculosis in surgically resected and autopsy lung specimens.

## 2. Materials and Methods

A descriptive retrospective and prospective histomorphological study of pulmonary tuberculosis was undertaken in the Department of Pathology, Kasturba Medical College, Mangalore, India, over a period of 4 years from 2008 to 2011. The specimens comprised of 33 autopsy cases in which sections from both lungs were submitted and 7 surgically resected cases including 4 lobectomy, 2 pneumonectomy, and 1 lung tissue for frozen section. Lung biopsies were excluded. Information regarding each patient was obtained from medical records or autopsy files. The specimens were received in 10% formalin. Gross examination was performed and representative sections were submitted. Slides were stained with Haematoxylin and Eosin (H&E) along with Ziehl-Neelsen (ZN) stain for identification of tuberculous bacilli.

The criteria for acceptance of a case as tuberculosis included the following:

- Demonstration of acid fast bacilli on ZN stain.
- Presence of necrotizing granulomatous inflammation.
- In absence of caseous necrosis or failure to demonstrate acid fast bacilli, past history of tuberculosis with response to treatment with antituberculous therapy.
- Exclusion of other causes of granulomatous inflammation.

Fungal infections and other noninfectious causes of granulomatous diseases were excluded by careful microscopic examination and application of special stains wherever required.

The histomorphological pattern noted the distribution of the lesions and the morphologic features of the tissue reaction. Other nonneoplastic findings in adjacent lung parenchyma were noted as well.

## 3. Results

The material for the study comprised 40 cases diagnosed as pulmonary tuberculosis on autopsy and resected lung specimens. Their age ranged from 20 to 75 years with a mean age of 41 years; the majority of the cases were in the 3rd decade (40%). There was a male predominance (92.5%).

The total number of autopsies performed during this period was 3034. Thus, frequency of tuberculosis in local autopsy service was 1.08%.

Prior to autopsy, a clinical diagnosis of pulmonary tuberculosis was known in only 4 of 33 (12.1%) cases. The majority of cases had presented with sudden death (19; 57.6%); 10 cases brought dead to mortuary were destitute; in these cases it was presumed that the death was sudden since they did not seek medical advice for their illness leading to death. These were subsequently diagnosed on histopathology as disseminated tuberculosis (4 cases), fibrocavitary tuberculosis (2 cases), and fibrocaseous tuberculosis (4 cases). Associated pneumonic changes were observed in 4 cases. The remaining 9 admitted patients with sudden death had disseminated TB (6 cases), fibrocaseous tuberculosis (2 cases), and tubercular bronchopneumonia (1 case). These cases had rapid deterioration of symptoms upon admission. Additional finding of left ventricular subendocardial ischemia with block of left coronary artery in a case of disseminated tuberculosis and healed myocardial infarction was observed in a case of fibrocaseous tuberculosis. Death in other cases was attributed to acute respiratory distress syndrome (ARDS) (4; 12.1%), lung abscess (2; 6.0%), and one case each with poisoning, drowning, complicated malaria, and a known diabetic with alcoholic cirrhosis. One patient with ARDS was coinfected with HIV.

Seven cases were operated upon; 2 cases were operated upon due to suspicion of malignancy. The remaining 5 cases were known cases of pulmonary tuberculosis with history of antitubercular treatment (ATT) and associated complications, namely, hemoptysis, empyema, bronchiectasis, and aspergilloma. Resistance to ATT was noted in one case which revealed positive acid fast bacilli in spite of regular treatment and presented with empyema.

On gross examination, pleural thickening was observed in 30 cases. The cut surface revealed caseation, consolidation, and miliary nodules in 10 cases each, cavity in 5 cases, and dilated bronchi, purulent exudates, and calcification in two cases each. Additionally, congestion and hemorrhage were observed in 8 cases and frothing was observed in 2 cases.

**Figure 1:**
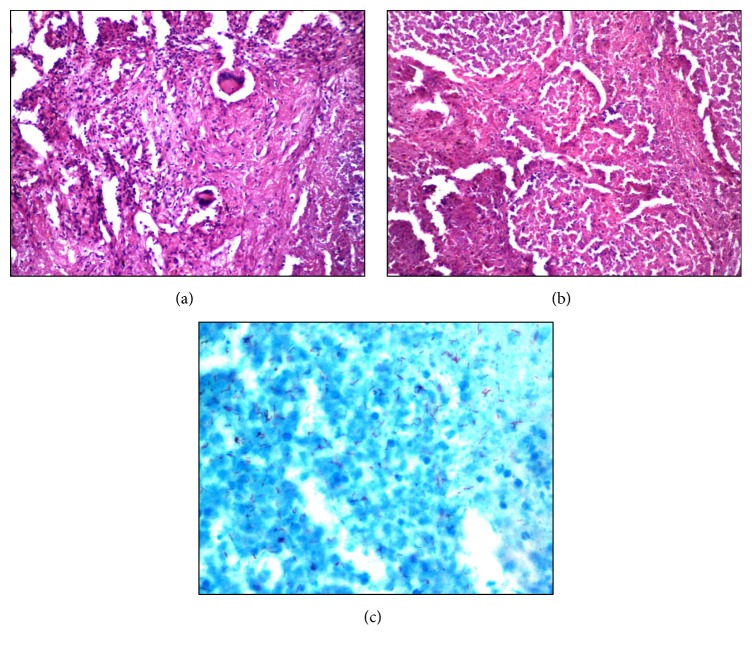
Microscopy examination. (a) Epithelioid granuloma with multinucleated giant cells (H&E, ×400). (b) Necrotic acute inflammatory exudate in alveolar spaces (H&E, ×400). (c) Numerous acid fast bacilli (ZN stain, ×1000).

The histological evaluation of lung sections (Figure 1) revealed necrotizing granulomas in 32 cases (80%), nonnecrotizing granulomas in 2 cases (5%), caseous necrosis with few Langhans giant cells in 2 cases (5%), and only caseous necrosis in 4 cases (10%).

ZN stain for acid fast bacilli (AFB) was positive in 57.5% of cases including 21 of 33 (63.6%) autopsy cases and 2/7 (28.5%) surgically resected specimens. All these cases showed presence of caseous necrosis on H&E stain.

Treatment history was not available in 12 cases that were brought dead to mortuary; 8 cases including 6 destitute and one case each of poisoning and drowning showed AFB positivity. The remaining were hospital based autopsies, with deaths due to ARDS in 4 cases (all were positive for AFB), lung abscess in 2 cases (AFB positivity in both cases), and unexplained sudden death in 9 cases (AFB positivity in 5 cases). Four were diagnosed cases of pulmonary tuberculosis with history of ATT treatment (one of these patients was positive for AFB). One case was admitted with complicated malaria (AFB negative) and one case was diabetes mellitus with alcoholic cirrhosis (positive for AFB). Among the 7 cases which were operated upon, acid fast bacilli was observed in two cases, including a case with clinical suspicion of malignancy revealing necrotizing granulomatous inflammation with consolidation and another case of empyema associated with fibrocaseous tuberculosis.

16 cases (37.5%) of pulmonary tuberculosis were also associated with other nonneoplastic findings in adjacent lung parenchyma or pleura, with acute bronchopneumonia constituting the most common finding followed by empyema, bronchiectasis, and aspergilloma (Table 1). In 10 cases dissemination to other organs was observed including liver in 9 cases, spleen in 8 cases, and kidney and brain in one case each. Hilar lymph nodes were involved in three cases.

**Figure 2:**
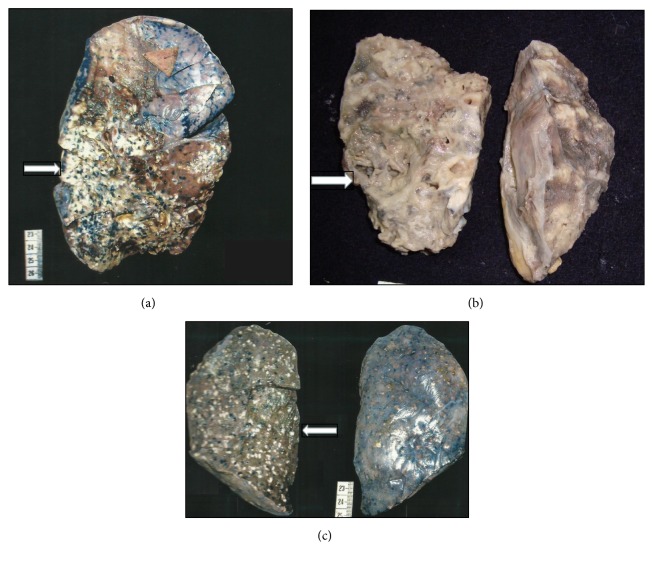
Histomorphological patterns on gross examination of lung (a), fibrocaseous tuberculosis (b), fibrocavitary tuberculosis with bronchiectasis, and (c) multiple millet sized tubercles in miliary tuberculosis.

Histopathological pattern analysis revealed primary caseating tuberculosis with involvement of hilar lymph nodes in 3 cases. Postprimary tuberculosis was seen in form of fibrocavitary, fibrocaseating, and tubercular bronchiectasis in 14 cases. Tubercular consolidation, miliary tuberculosis, and empyema were reported in 23 cases; these findings can be a manifestation of both of the above patterns. Tubercular bronchopneumonia was seen associated with fibrocavitary, fibrocaseous, or disseminated tuberculosis reflecting the fact that these finding were associated with postprimary tuberculosis. Miliary tuberculosis was observed especially in patients presenting with dissemination to other organs (Table 2) (Figure 2).

## 4. Discussion 

TB is an infectious disease caused by the bacillus* Mycobacterium tuberculosis*. It typically affects the lungs (pulmonary TB) but can affect other sites as well (extrapulmonary TB). It is more common among men and affects mostly adults in economically productive age groups. The latest estimates included in global tuberculosis report in 2013 were 8.6 million new TB cases in 2012 and 1.3 million deaths due to tuberculosis. The number is unacceptably large especially that deaths can be prevented with access to health care and proper treatment [9]. The present study highlights the magnitude of undetected pulmonary tuberculosis diagnosed at autopsy and of patients presenting with complications/sequelae of the disease.

Aziz et al. [10] in a study on 46 cases of tuberculosis of lung observed a bimodal distribution with a younger age group of less than 30 years and an older age group of more than 50 years. In our study most of the patients were young, in the third decade of life with male predominance.

In India, the Revised National Tuberculosis Programme detects nearly three times more male than female TB patients [11]. In a study conducted in similar region Ganapathy et al. [12] observed a male predilection. The reasons for this are unclear. It could be due to epidemiological differences, exposure to risk of infection, and progression from infection to disease. Due to various stigmata and sociocultural factors associated with this disease, women in developing countries confront more barriers than men in accessing health care services.

Combination drug therapy is the first-line of treatment for pulmonary tuberculosis. Surgery for tuberculosis has been of major concern because of its associated morbidity and mortality [4]. The common complications leading to surgery fall into three main categories: persistent or drug-resistant disease, bronchiectasis, and hemoptysis [13]. Similar findings were observed in the present study.

Tuberculous empyema represents a chronic, active infection of the pleural space that contains a large number of tubercle bacilli. Treatment consists of pleural space drainage and antituberculous chemotherapy. Problematic treatment issues include the inability to reexpand the trapped lung and difficulty in achieving therapeutic drug levels in pleural fluid, which lead to drug resistance. Surgery too is often challenging [14]. Bhattacharya et al. [15] observed tuberculosis to be the cause of bilateral empyema in 58.8% cases.

Bronchiectasis is one of the commonest abnormalities seen in pulmonary tuberculosis. It is rarely gross and is found in the presence of secondary advanced parenchymatous disease [16]. The exact incidence of bronchiectasis as sequela to tuberculosis is difficult to determine as studies are done on selected groups and varies in developed and developing countries [17]. In an Indian study, Sahoo et al. [18] observed 62% cases with posttubercular bronchiectasis.

Fibrocavitary tuberculosis with aspergillosis in right upper lobe was diagnosed in a case. Aspergilloma represents the most common infectious complication of pulmonary tuberculous cavities. A combination of antitubercular drug treatment with surgical resection is the treatment of choice [19].

Two cases were operated upon with suspicion of malignancy. Frozen section was done in one case; while in the other case lobectomy was performed. Both showed necrotizing granulomas with associated pneumonic consolidation. Pulmonary tuberculosis can present with a variety of radiological findings. Tuberculoma and focal organizing pneumonia can mimic neoplasm because of similar radiological findings on plain chest radiographs and CT scans. Also, diffuse form of bronchoalveolar carcinoma can appear radiologically as air space consolidation resembling pneumonia, multiple nodules mimicking disseminated tuberculosis, fungal infection, metastasis or as ill-defined masses, cavitating nodules, or pleural effusions [20–22]. In such cases final diagnosis is made on histopathology.

Clinically undiagnosed TB constitutes a substantial proportion of active TB cases diagnosed at autopsy. In the present study 87.8% of cases were diagnosed on autopsy. These undetected cases at autopsy range from 44% to 70% in various studies [6, 23–25].

In the present study 57.6% had presented with sudden death. Rastogi et al. [5] observed tuberculosis as the most important cause of sudden unexpected death involving respiratory system in 61.64% and it contributed to 16.42% of total sudden deaths. This increase in the frequency of sudden death due to TB despite better facilities is a cause of concern.

Reddy et al. [26] in a study on patterns of tuberculosis in autopsy cases observed that the commonest type of lesion in the lung was fibrocaseous variety (66.56%). Miliary lesions were seen in 22.7% whereas the pneumonic type was seen in 10.74%. In the present study most of the cases were of tuberculous consolidation (27.5%) followed by miliary tuberculosis and fibrocaseous TB (25% and 17.5%, resp.).

Rarely pulmonary tuberculosis may present as an acute pneumonia with respiratory failure. TB misdiagnosed as community acquired pneumonia (CAP) accounts for approximately 35% of microbiologically confirmed pneumonias. Tuberculous consolidation was present in 27.5% of cases in the present study. The incidence of acute respiratory failure is 1.5% among patients hospitalized with pulmonary TB. In the present study 12.1% of deaths were due to ARDS. This acute presentation may result from primary infection, progressive primary disease, reactivation of latent TB, or atelectasis [27].

Miliary TB is a potentially fatal form of TB that results from massive lymphohematogenous dissemination. Diagnosis can perplex even the experienced because of nonspecific symptoms and chest radiographs do not always reveal classical miliary changes. Atypical presentations like cryptic miliary TB and ARDS further lead to delay in diagnosis. It is characterized by high mortality rate, despite the availability of effective treatment [28].

The usual histologic reaction in TB is a granulomatous inflammation with varying numbers of accompanying nonnecrotizing granulomas [29]. Necrotizing granulomas were observed in 80% cases in present study. Similar findings were observed by Das et al. [30]. They observed necrotizing granulomas in 44.7%, nonnecrotizing granuloma in 10.5%, and only caseous necrosis in 44.7% cases.

Granulomatous diseases of lung often present a diagnostic challenge. The diagnosis requires familiarity with the tissue reaction as well as with the morphologic features of the organisms, including appropriate interpretation of special stains. Nonneoplastic granulomatous diseases like Wegener's granulomatosis and Churg-Strauss syndrome microscopically show necrotizing granulomas with features of vasculitis. Granulomas of tuberculosis may also involve blood vessels resembling true primary vasculitic disorder posing a diagnostic difficulty. However, in the present study, involvement of blood vessels was not seen. Many infectious granulomatous lesions may also mimic tuberculosis. Fungal infections especially histoplasma show necrosis similar to tuberculosis. Clue to diagnosis is careful interpretation of surrounding tissue reaction and special stains [31].

Acid fast bacilli (AFB) positivity in smears and histological specimens depends on the bacillary load of the specimen and the type of the material [32]. Acid fast bacilli positivity was seen in 57.5% in the present study. Similarly, Park et al. [33] observed microbiologically confirmed pulmonary tuberculosis in 50% of patients. The bacilli are normally scanty in tuberculous tissue and their identification with ZN stain requires careful examination. Tissue containing necrotizing granulomas is more likely to give positive results than specimens showing only nonnecrotizing granulomas, poorly formed granulomas, or acute inflammation. Failure to demonstrate them does not exclude a diagnosis of tuberculosis. Making a definitive diagnosis in the future may incorporate application of immunohistochemistry or molecular techniques such as polymerase chain reaction to paraffin sections [34].

As the present study included mostly autopsy cases it was subject to various limitations. The complete details of underlying risk factors such as coinfection with HIV, steroid therapy, and BCG exposure were not available. Only one case was known to be infected with HIV. Tuberculosis associated with HIV infection is aggressive and is characterized by widespread dissemination. These patients are susceptible to rapidly progressive tuberculosis. Recurrence of tuberculosis may occur after successful treatment due to exogenous reinfection. The risk of developing tuberculosis disease in HIV-infected patients in India is estimated to be 7 cases per 100 person-years at risk [34, 35].

Also, clinical data or test for confirmation of multidrug-resistant (MDR) tuberculosis was not available in these cases. AFB was positive in a patient on ATT who developed empyema for which he was operated upon and in one patient who died during the course of disease for which autopsy was done. These cases may reflect probable resistance, reactivation, or reinfection.

In conclusion, pulmonary tuberculosis is one of the oldest known diseases with rapidly increasing newer diagnostic modalities and treatment strategies. But still the incidence of disease has not decreased. Histopathology remains one of the important methods for diagnosis. Tuberculosis if diagnosed in time is treatable and patient may get cured without any complications. However, if the patient is immunocompromised or elderly or presents in late stages of the disease or unusual presentations leading to delayed diagnosis, they may have a fulminant course with bilateral involvement and respiratory distress. The morbidity and mortality are high in this population of cases. Undiagnosed tuberculosis may be a health hazard to the public and healthcare providers. These cases form a substantial number in autopsy studies, probably representing the tip of the iceberg and indicating seriousness of the problem of tuberculosis.

Protective strategies like risk assessment, early diagnosis, recognition of lesions, use of methods to reduce infection transmission, and effective regular treatment are advocated to curtail the spread of tuberculosis [5, 19].

## Figures and Tables

**Table 1:** Various pulmonary diseases associated with tuberculosis.

TB with other diseases	Cases	Percentage
Acute bronchopneumonia	11	68.7
Empyema	02	12.5
Bronchiectasis	02	12.5
Aspergilloma	01	06.3

Total	16	100

**Table 2:** Histopathological pattern of tubercular lesions in lungs.

Final diagnosis	Cases	Percentage
Tubercular consolidation	11	27.5
Miliary tuberculosis	10	25.0
Fibrocaseous tuberculosis	07	17.5
Fibrocavitary tuberculosis	05	12.5
Caseating tuberculosis	03	7.5
Tubercular empyema	02	5.0
Tubercular bronchiectasis	02	5.0

Total	40	100
